# Patterns in Patient Access and Utilization of Online Medical Records: Analysis of MyChart

**DOI:** 10.2196/jmir.8372

**Published:** 2018-02-06

**Authors:** Donald A Redelmeier, Nicole C Kraus

**Affiliations:** ^1^ Department of Medicine University of Toronto Toronto, ON Canada; ^2^ Sunnybrook Hospital University of Toronto Toronto, ON Canada

**Keywords:** electronic health records, personal health information, patient portals, shared decision making, doctor patient relationship

## Abstract

**Background:**

Electronic patient portals provide a new method for sharing personal medical information with individual patients.

**Objective:**

Our aim was to review utilization patterns of the largest online patient portal in Canada's largest city.

**Methods:**

We conducted a 4-year time-trend analysis of aggregated anonymous utilization data of the MyChart patient portal at Sunnybrook Health Sciences Centre in Ontario, Canada, from January 1, 2012, through December 31, 2015. Prespecified analyses examined trends related to day (weekend vs weekday), season (July vs January), year (2012 vs 2015), and an extreme adverse weather event (ice storm of December 20-26, 2013). Primary endpoints included three measures of patient portal activity: registrations, logins, and pageviews.

**Results:**

We identified 32,325 patients who registered for a MyChart account during the study interval. Time-trend analysis showed no sign of attenuating registrations over time. Logins were frequent, averaged 734 total per day, and showed an increasing trend over time. Pageviews mirrored logins, averaged about 3029 total per day, and equated to about 5 pageviews during the average login. The most popular pageviews were clinical notes, followed by laboratory results and medical imaging reports. All measures of patient activity were lower on weekends compared to weekdays (*P*<.001) yet showed no significant changes related to seasons or extreme weather. No major security breach, malware attack, or software failure occurred during the study.

**Conclusions:**

Online patient portals can provide a popular and reliable system for distributing personal medical information to active patients and may merit consideration for hospitals.

## Introduction

Online patient portals are an innovation for communicating medical information to patients in a reliable, detailed, quick, convenient, and secure manner. The information typically includes blood test results, medical imaging reports, and medical consultation notes. Through portals, patients can track their own information to promote self-management, review past trends, refresh fallible memory, digest overwhelming material, and share records with other health care professionals at separate institutions [1,2]. A patient who becomes sick while vacationing, for example, can use an online patient portal to retrieve their usual creatinine level, prior echocardiogram, or current medication list to help inform care for an emergency occurring in a foreign land [3,4]. In theory, these patient portals might also decrease health care costs by avoiding duplications of services [5].

The literature provides a mixed picture of whether patient portals improve medical outcomes. One study of diabetic patients randomized to an enhanced program including an online patient portal found a significant improvement in glycemic control measured as HbA1c after one year (7.3% vs 8.1%, *P*=.003) [6]. This finding replicated in some studies and failed to replicate in other studies [7,8]. Further research on patients with breast cancer, human immunodeficiency virus (HIV) infection, renal insufficiency, or heart failure have shown increases in patient satisfaction but equivocal changes in clinical outcomes [9]. The latest systematic reviews find no consistent objective benefit attributable to online patient portals and conclude that more research is needed [10-12]. The overall modest results in medicine differ substantially from banking or other commercial industries that profit greatly from online customer access [13].

The absence of rigorous evidence has undermined the enthusiasm toward online patient portals for hospitals and health care systems [14]. Most projects are in their infancy and motivated by general regulations based on theoretic predictions. Furthermore, most predictions lack real-world data about utilization, reliability, and popularity. Developers also tend to be skilled in information technology yet lacking in medical experience. In this paper, we review the performance of the largest online patient portal in Canada’s largest city (Toronto). The intent is to provide baseline data, trends, and patterns of utilization for others planning to create or to evaluate a new online patient portal. We do not examine technical points on software development, and we focus on relatively mature years of operation after completing initial pilot testing.

## Methods

### Study Setting

Sunnybrook Health Sciences Centre is a major academic hospital in Toronto, Canada. Toronto is a city of 6 million individuals and the fourth largest urban area in North America. In a typical year during the study, Sunnybrook had over 600 acute care beds, 35,000 admissions, 60,000 emergency cases, 500,000 outpatient visits, 16,000 surgical operations, and 4000 newborn deliveries (total exceeding 600,000 patients annually) [15,16]. Sunnybrook is one of five general hospitals affiliated with the University of Toronto, funded under the universal health insurance of the Ontario government, staffed by more than 10,000 health care professionals including volunteers, and run with a budget of Can $1 billion annually [17]. For perspective, the Ontario region had a population of 14 million individuals, 155 hospitals distributed over 238 sites, and 213 acute care emergency departments [18,19].

### MyChart System

The patient portal (named MyChart, not to be mistaken with Epic MyChart) was developed by Sunnybrook in 2005, had an early launch in 2006, became widely available in 2007, and later expanded to 10 other institutions [20,21]. The core functions provided patients read-only access to blood test results, imaging reports, and physician notes. Additional features later developed included access to clinic schedules, pathology reports, microbiology data, electrocardiogram results, and patient diaries. The MyChart system did not support secure messaging, patient annotation, prescription renewals, or targeted education (presumably, patients obtain such services through other channels). A MyChart account is available at no cost to patients, does not require a clinician’s consent, can be accessed by the Internet, and has a standard interface (Multimedia Appendix 1).

### Past Publications of Sunnybrook MyChart System

The Sunnybrook MyChart system has been subjected to early analyses and small studies. The median user was a middle-aged woman who lived within 100 km of the hospital [22]. Individual patient demographic profiles varied substantially and spanned a wide range of diagnoses. Most patients were ambulatory, had at least one chronic medical disease, and expressed a particular interest in accessing their laboratory test results [23]. Patients had originally learned of Sunnybrook MyChart from word-of-mouth, brochures, or posters in high-traffic areas of the hospital and subsequently taught themselves how to use it without a formal Sunnybrook training program [24]. About half shared their personal access identifier and password with at least one family member. Some Sunnybrook physicians did not immediately embrace the initiative for this patient portal.

### Sample Selection

We identified the most recent 4 consecutive calendar years of utilization data for the Sunnybrook MyChart system encompassing all complete years of system information (January 1, 2012, to December 31, 2015). This selection interval excluded the initial years of system development, early testing, and major upgrades that preceded widespread launch (April 1, 2007, to December 31, 2011). The analyses focused on aggregate traffic details to respect medical privacy and removed identifiable information to preserve patient confidentiality. This analytic strategy also included patterns where an individual patient might have shared their account details with family or other trusted delegates. The study protocol was approved by the Research Ethics Board of the Sunnybrook Research Institute including a waiver for patient consent on the condition that the study examined only aggregated anonymous utilization data.

### MyChart Registration, Login, and Pageviews

Individual patients obtained a MyChart account by submitting a simplified application with personal identification for authentication (denoted as a MyChart registration). The onsite contact office for handling submissions was located adjacent to the hospital cafeteria and delivered as an on-demand service with no appointment. MyChart registration provided patients a temporary password, instructions for website navigation, and access to an unlimited number of website visits (denoted as a MyChart login). In turn, each MyChart login allowed an unlimited number of connections to individual features (denoted as a MyChart pageview). A MyChart pageview could contain differing amounts of information; for example, a single pageview of laboratory results could encompass dozens of individual test results.

### Aggregated MyChart Traffic Information

The MyChart system routinely tracked activity as part of system monitoring, quality assurance, and ongoing maintenance. These aggregated data were automatically indexed to time and available in an aggregated manner grouped as consecutive days with no exclusions and no missing data. By design, the aggregated data were stripped of individual patient identifiers and expunged of actual detailed demographic or medical information. The purpose was to maintain full patient privacy and data security yet still provide health service research data necessary for evaluating comprehensive patterns of utilization (denoted as MyChart traffic information). MyChart traffic information is not traceable to individual patients, does not reveal individual medical characteristics, and does not breach doctor-patient confidentiality.

### Statistical Analysis

The primary analysis used time-trend analysis to describe MyChart traffic information over the study interval. Separate analyses were conducted for each of the primary measures of MyChart activity, specifically, registrations, logins, and pageviews. Secondary analyses examined specific priority pageviews of blood test results, medical imaging reports, and medical consultation notes. We conducted comparative analyses to also examine daily trends (contrasting weekends of Saturdays and Sundays), seasonal trends (focusing on January and July), annual trends (highlighting the first and final year of observation), and resiliency to unforeseen shocks (evaluating the 7-day Toronto ice storm of December 20-26, 2013) [25,26]. The unit-of-analysis throughout was the individual day to avoid small cell sizes and allow adequate statistical power.

## Results

**Figure 1:**
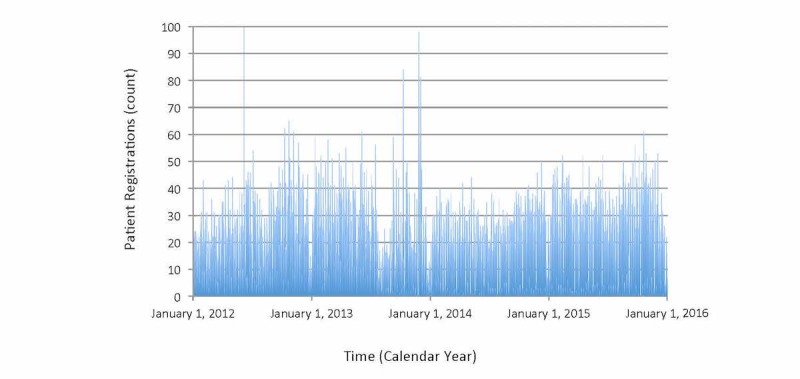
Time trend of MyChart registrations (x axis=time in consecutive days spanning 4 total years, y axis=total number of patient registrations occurring on corresponding day). Main findings show high variability with no major increasing or decreasing trend over time; slight regular reduction in late December also evident for each year.

A total of 32,325 patients received a MyChart registration during the 4-year study interval (total of 8081 patients annually). This equaled 22 registrations during the average day, 825 registrations during the most active day, and 0 registrations during the least active day (Figure 1). On average, fewer registrations occurred on weekends than weekdays (6 vs 29, *P*<.001), the month of July was similar to January (20 vs 22, *P*=.374), and the first year was similar to the final year (24 vs 24, *P*=.825). The Toronto ice storm led to an equivocal 61% reduction in registrations compared to the same dates in other years (4 vs 11, *P*=.089). Time trends showed a complicated profile, possibly related to fluctuating interest or promotion campaigns, with no significant sign of attenuating enrollment over time (Figure 1).

**Figure 2:**
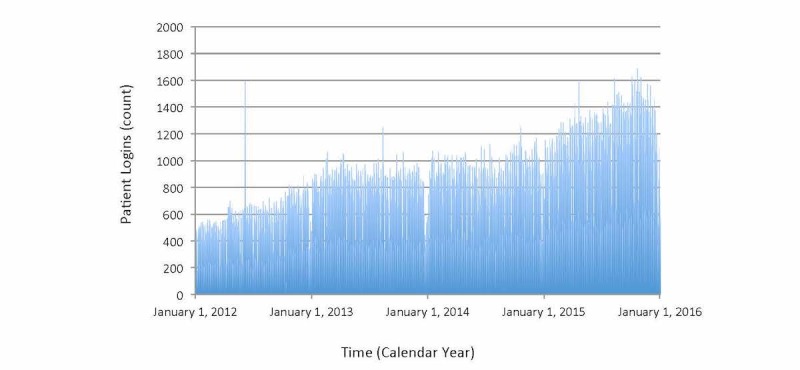
Time trend of MyChart logins (x axis=time in consecutive days spanning 4 total years, y axis=total number of patient logins occurring on corresponding day). Main findings show daily average of about 1000 with substantial increasing trend over time and slight reductions in late December also evident for each year. Anomalous spike on June 5, 2012, visible and unexplained, and no anomaly visible on June 6, 2012.

MyChart logins were frequent and increased substantially during the study interval (see Table 1). This equaled 734 logins during the average day, 1687 logins during the most active day, and 87 logins during the least active day (Figure 2). On average, weekends were less active than weekdays (385 vs 873, *P*<.001), the month of July was slightly more active than January (735 vs 660, *P*=.045), and the first year was substantially less active than the final year (486 vs 1013, *P*<.001). The Toronto ice storm led to an equivocal reduction in logins (367 vs 564, *P*=.89). Time trends showed a generally increasing profile, with a single unexplained anomaly on June 5, 2012.

**Figure 3:**
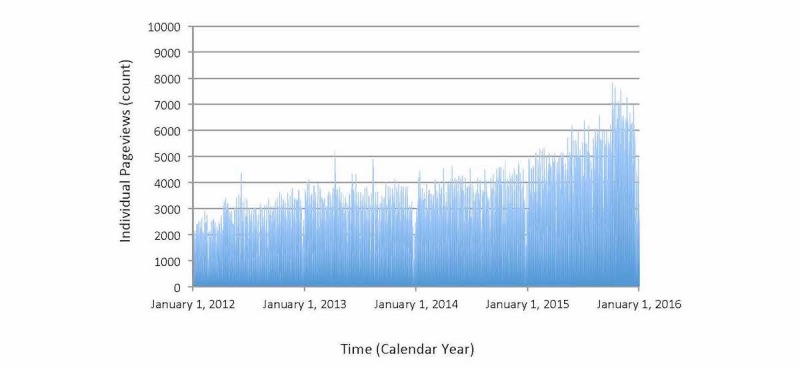
Time trend of MyChart pageviews (x axis=time in consecutive days spanning 4 total years, y axis=total number of pageviews occurring on corresponding day). Main findings show daily average of about 5000 with substantial increasing trend over time and slight reductions in late December also evident for each year. Anomalous spikes on Jun. 6, 2012, Apr. 10, 2013, Aug. 13, 2013, and Oct. 14, 2015, visible and unexplained.

**Figure 4:**
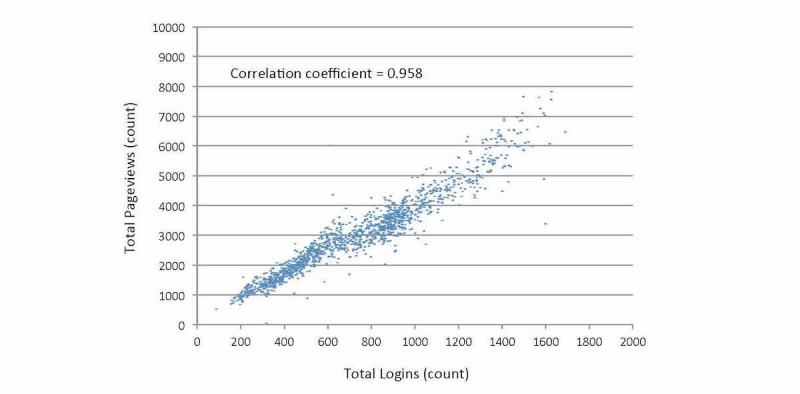
Scatterplot correlating logins and pageviews (x axis=total number of logins occurring on consecutive days over 4 total years, y axis=total number of pageviews occurring on corresponding day). Main findings show tight correlation that equals about 4-5 pageviews per login; slight anomalies also evident as outlier points that remain unexplained.

MyChart pageviews tended to mirror MyChart logins with a relatively stable ratio equal to about 5 pageviews for the average login. This equaled 3029 pageviews during the average day, 7823 pageviews during the most active day, and 48 pageviews during the least active day (Figure 3). On average, weekends were less active than weekdays (1714 vs 3554, *P*<.001), the month of July was marginally more active than January (2987 vs 2768, *P*=.133), and the first year was substantially less active than the final year (2197 vs 4254, *P*<.001). The Toronto ice storm led to a 55% reduction in pageviews (1065 vs 2380, *P*=.010). Time trends showed a generally increasing profile with no major anomalies. The ratio of pageviews to logins was remarkably consistent (Figure 4).

**Table 1:** Summary of access to online patient portal by year.

Activity	2012, n	2013, n	2014, n	2015, n
Registrations	8909	7891	6837	8688
Logins	177,793	251,885	272,494	369,764
Pageviews	804,042	978,302	1,090,545	1,552,544

Specific MyChart features varied substantially in activity. The most frequent pageviews were clinical notes averaging about 28% of total pageviews. The next most popular pageviews were laboratory results (26%), medical imaging reports (17%), and appointment schedules (12%). In contrast, pathology notes (6%), electrocardiogram reports (4%), and microbiology findings (3%) received relatively few pageviews, perhaps due to their selected specialized nature (number of eligible relevant patients unknown). The lowest extreme was patient diaries (1%) , which received few pageviews despite being one of the few features that allowed patients self-input and self-expression. Time-trend analysis of specific pageviews showed no major anomalies during the study interval.

The distinctly low numbers of pageviews for patient diaries were subjected to further exploratory analyses as a possible indirect marker of patient engagement and self-management. The overall pattern equaled 17 diary pageviews during the average day, 1032 during the most active day (June 6, 2012), and 0 during the least active day. On average, weekends were less active than weekdays (10 vs 19, *P*<.001), July similar to January (15 vs 16, *P*=.149), and the first year similar to the final year (21 vs 17, *P*=.241). The Toronto ice storm led to no significant difference in diary pageviews (7 vs 11, *P*=.146). Together, the contrasts suggested that high volumes of MyChart activity were not matched by high volumes of this marker of patient engagement.

## Discussion

### Principal Findings

We studied the online patient portal at one large hospital to assess aggregated utilization over 4 years. We found that new registrations were uneven and amounted to about 1% of total patients treated at the hospital (8081/600,000). Despite the low uptake, total utilization grew substantially and exceeded 1000 logins daily in the final year. Utilization declined on weekends yet remained reliable despite an unforeseen extreme ice storm. Patients averaged 5 pageviews per login, showed relatively high interest in viewing clinical notes, and had relatively little interest in maintaining a diary [27,28]. Together, these data suggest that online patient portals can provide a popular and reliable system for distributing medical information to a self-selected subgroup of patients.

### Limitations

An important limitation of our study is the absence of detailed data linking patient characteristics, diagnoses, treatments, and medical outcomes: this limitation was necessary for the ethics safeguards to analyze patient activity without patient consent. Our study also did not distinguish how much utilization occurred from one patient with multiple logins versus multiple patients with one login each. The digital data, furthermore, do not track a patient’s experience to test how a recent medical encounter changes subsequent online portal utilization or further nuances that examine which pageviews tend to be viewed first and for how long. The ultimate positive benefits of online patient portals for increasing patient satisfaction, knowledge, motivation, equity, and health outcomes are all topics that remain for future research.

A second limitation relates to generalizability because the Sunnybrook system is not the epitome of patient portals. The user interface can be awkward for some patients with cognitive impairment or those unfamiliar with medical data [29]. The system lacks some functions relevant to ongoing patient care such as secure messaging and prescription medication renewals. The system also has few of the connections necessary for networking between hospitals or physicians outside the region. The background software provides no customization based on an individual’s past search history or tailoring to a patient’s unique profile. All patient portals involve design compromises between functionality, simplicity, reliability, and security [30-34] and are likely to evolve further in future years.

Another limitation of our study is the absence of data on possible adverse effects of communicating large amounts of personal health information to individual patients. One worry is that patients lack formal medical training, misinterpret innocuous items, and can be upset about inconsequential anomalies [35,36]. A related concern is that some patients may obsess about minutiae, repeatedly refresh for updated data, or attempt to embarrass health care providers in a deliberate strategy of one-upmanship [37]. The direct viewing of physician notes also allows patients to detect undiplomatic language, spelling mistakes, and other awkward professional lapses [38]. Ultimately, these medical portals empower patients and thereby shift the balance of power around a clinical encounter.

A related limitation is the lack of data on physician behavior since health care providers are aware of patient portals and may adjust their documentation accordingly. One reaction is to stifle candid collegial dialogue due to concerns about later misinterpretation by the patient [39,40]. Another reaction involves unnecessary clutter or auto-text inserted as a medico-legal defensive strategy [41,42]. A further reaction is for health care providers or families to focus excess attention on online data and neglect the patient’s symptoms, physical findings, or other changes found at the bedside, not online [43,44]. A comprehensive online patient portal, moreover, could be counterproductive for a patient diagnosed with a personality disorder who can immediately access a physician’s notes.

### Future Directions

The Sunnybrook online patient portal has strengths that justify future research. The system provides a flexible platform for patients to help integrate clinical services, enhance self-management, and potentially improve health outcomes. Patients can easily share their medical information with family members or community physicians for broader system integration [45]. Patients can also use the system to incorporate tracking tools for personal health status (eg, ambulatory blood pressure monitors) [46]. Patients have frequent opportunities to comment on their experiences through user surveys and, in turn, developers are integrated with the larger health care team to facilitate system advances [47]. All these benefits might increase further with ongoing system expansion. The sustained high rates of utilization found in this study therefore suggest potentially sufficient sample size and statistical power necessary for planning future research.

### Conclusions

The utilization of online patient portals observed in this study indicates the technology is popular for a small subgroup of patients who register [48,49]. If each pageview were assumed equal in value to a 50¢ payphone call, for example, the net utilization in this study would be worth Can $1 million annually. If uptake could increase to 20% of total patients and patterns persisted, furthermore, the net utilization might be worth Can $25 million annually (Can $10 billion if extrapolated to all of Canada). Regardless of assumed uptake, this study also implies such value can accrue for years without a major security breach or software failure. As the Internet continues to transform society and individuals grow increasingly comfortable online, more patients may demand information technology solutions to help access personal health data [50,51]. Other hospitals, therefore, might consider an online patient portal for future years.

## Appendix

Multimedia Appendix 1 Screenshots of MyChart online patient portal from user perspective.
