# Supplementary figures and images for: Patterns in Patient Access and Utilization of Online Medical Records: Analysis of MyChart
Source: J Med Internet Res. 2018 Feb 6;20(2):e43. doi: 10.2196/jmir.8372 (PMC5820458; doi:10.2196/jmir.8372)

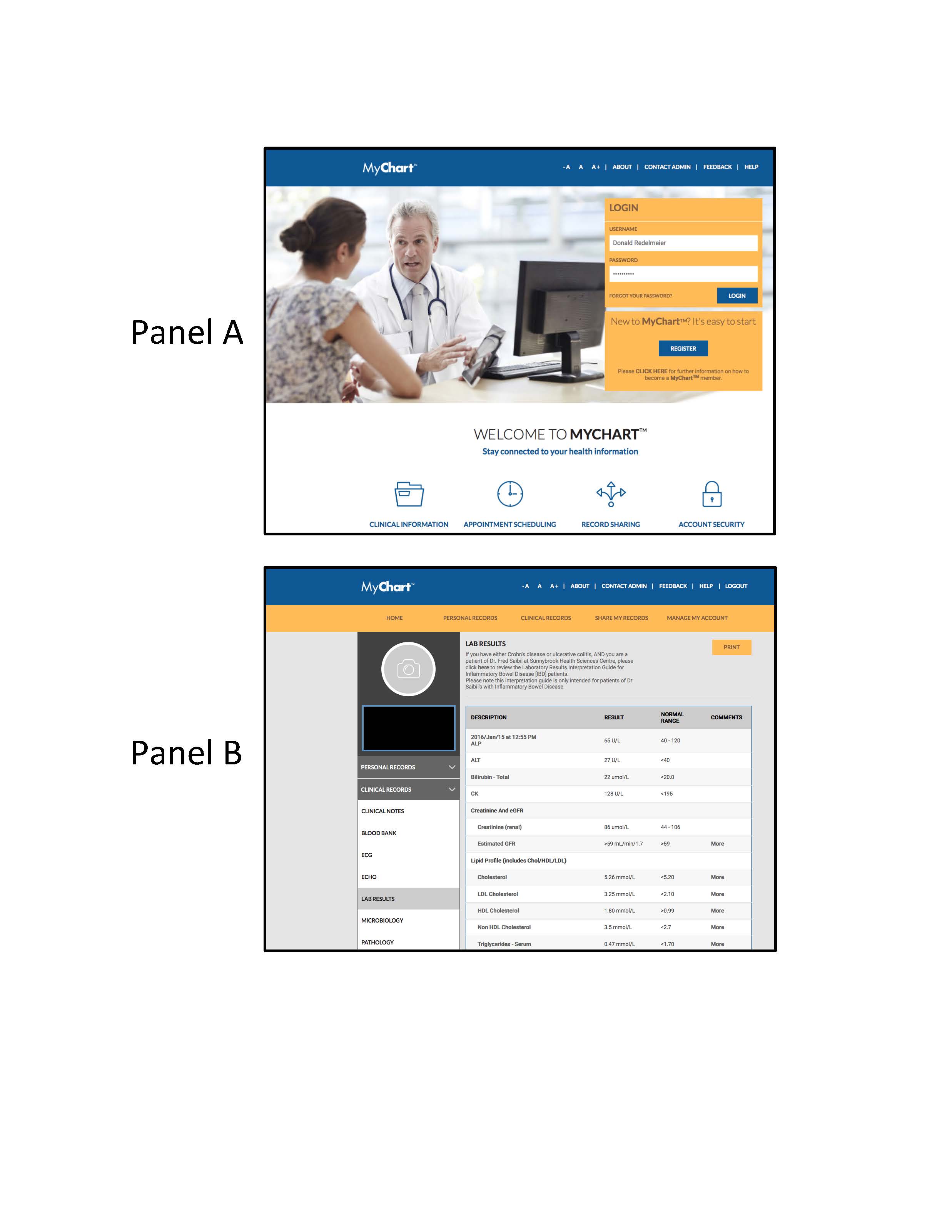

Supplement: Multimedia Appendix 1 [file jmir_v20i2e43_app1.jpg]
